# Floral Longevity of *Paphiopedilum* and *Cypripedium* Is Associated With Floral Morphology

**DOI:** 10.3389/fpls.2021.637236

**Published:** 2021-05-31

**Authors:** Feng-Ping Zhang, Jing-Qiu Feng, Jia-Lin Huang, Wei Huang, Xue-Wei Fu, Hong Hu, Shi-Bao Zhang

**Affiliations:** ^1^Yunnan Key Laboratory of Dai and Yi Medicines, College of Traditional Chinese Medicine, Yunnan University of Chinese Medicine, Kunming, China; ^2^Key Laboratory of Economic Plants and Biotechnology, Yunnan Key Laboratory for Wild Plant Resources, Kunming Institute of Botany, Chinese Academy of Sciences, Kunming, China; ^3^University of Chinese Academy of Sciences, Beijing, China; ^4^Yuxi Normal University, Yuxi, China

**Keywords:** *Cypripedium*, floral anatomy, floral longevity, floral water economy, functional traits, *Paphiopedilum*

## Abstract

Floral longevity (FL) is an important trait influencing plant reproductive success by affecting the chance of insect pollination. However, it is still unclear which factors affect FL, and whether FL is evolutionarily associated with structural traits. Since construction costs and water loss by transpiration play a role in leaf longevity, we speculated that floral structures may affect the maintenance and loss of water in flowers and, therefore, FL. Here, we investigated the slipper orchid *Paphiopedilum* and *Cypripedium*, which are closely related, but strongly differ in their FL. To understand the evolutionary association of floral anatomical traits with FL, we used a phylogenetic independent comparative method to examine the relationships between 30 floral anatomical traits and FL in 18 species of *Paphiopedilum* and *Cypripedium*. Compared with *Paphiopedilum* species, *Cypripedium* species have lower values for floral traits related to drought tolerance and water retention capacity. Long FL was basically accompanied by the thicker epidermal and endodermal tissues of the floral stem, the thicker adaxial and abaxial epidermis of the flower, and low floral vein and stomatal densities. Vein density of the dorsal sepals and synsepals was negatively correlated with stomatal density. Our results supported the hypothesis that there was a correlation between FL and floral anatomical traits in slipper orchids. The ability to retain water in the flowers was associated with FL. These findings provide a new insight into the evolutionary association of floral traits with transpirational water loss for orchids under natural selection.

## Introduction

Floral longevity (FL) is an important floral functional trait that influences the reproductive success of flowering plants by affecting the chance of insects visiting flowers (Primack, 1985; Ashman and Schoen, 1994). Similar to other floral traits, FL shows great diversity in angiosperms. For example, the flowers of *Ipomoea* and *Oenothera* species remain open for only a few hours (Kerner von Marilaun, 1895), whereas the flowers of other species, such as orchids, can last for weeks or even several months (Kerner von Marilaun, 1895; Zhang et al., 2017). FL may be affected by biotic and abiotic factors (Yasaka et al., 1998; Arathi et al., 2002; Rathcke, 2003; Harder and Johnson, 2005; Vespirini and Pacini, 2005; Itagaki and Sakai, 2006; Weber and Goodwillie, 2012; Arroyo et al., 2013; Jorgensen and Arathi, 2013). For example, pollination can shorten FL (Giblin, 2005; Weber and Goodwillie, 2012). In addition, the length of FL is closely related to water-use efficiency, altitude, and temperature (Vespirini and Pacini, 2005; Arroyo et al., 2013; Jorgensen and Arathi, 2013). To maintain the normal display and physiological metabolism of flowers, sufficient water and energy are very important. Previous studies have found that water plays an important role for bud expansion, flower opening, and nectar production during the whole stage of flower display (Mohan Ram and Rao, 1984; Patino and Grace, 2002; Tsukaguchi et al., 2003; van Doorn and van Meeteren, 2003; Galen, 2005). In cut flower display, fungicides can be added to the preservative solution to reduce the blockage of microbial breeding on flower vessels to prolong the vase life, and floral stems can be re-cut under water to maintain the continuity of the water column and the water transport in flowers, therefore extending FL (Lu, 2006; Guo, 2013; Li et al., 2020). Although water balance plays an important role in the maintenance of flower life span and turgor pressure, it is still unclear which flower traits affect the water balance and FL.

Flowers contribute only slightly to carbon assimilation of the whole plant and have relatively shorter life span than leaves, but they may still transpire a large amount of water during the flowering period (Lambrecht et al., 2011; Roddy and Dawson, 2012; Lambrecht, 2013; Teixido and Valladares, 2014). However, flowers in water-deficit conditions may wilt, which may affect pollination success. Thus, flowers must keep their turgidity and water balance, maintaining floral functions to attract pollinators (Lambrecht et al., 2011; Zhang et al., 2017). Although long-lived flowers may increase the probability of successful pollination, in particular when pollinators are scarce, they may also represent a major drain on the water budget of the plant (Nobel, 1977; Rathcke, 2003; Jorgensen and Arathi, 2013; Zhang et al., 2017).

Previous studies have found that leaf anatomical traits play an important role in maintaining the leaf life span in *Paphiopedilum* and *Cypripedium* orchids (Chang et al., 2011; Guan et al., 2011; Yang et al., 2018). For example, a high degree of leaf succulence can contribute to greater water storage capacity, which is beneficial under conditions of drought (Guan et al., 2011; Zhang et al., 2012, 2015; Yang et al., 2018). Leaf anatomy plays a key role in maintaining the water balance in *Cymbidium* species (Zhang et al., 2015). Traits, such as epidermis thickness, stomatal density, and stomatal size, are usually regarded as the adaptations to water-limited conditions, since they contribute to reduce water transpiration rate from leaves (Haworth and McElwain, 2008; Mill and Stark Schilling, 2009; Zhang et al., 2012). Despite this functional link between leaf longevity and morphology has been confirmed, the evolutionary association between the longevity and floral structural traits is unknown.

The Orchidaceae is one of the most diverse families of flowering plants in terms of floral form, size, color, and fragrance, with approximately 28,000 species and 763 genera (Crain and Tremblay, 2014; Christenhusz and Byng, 2016; Zhang S. B. et al., 2018). Most orchids grow on the forest canopies where pollinating insects are relatively rare, but they rely on insects for pollination. In addition, due to the unique floral structure of orchids, many have a special lock–key relationship with pollinating insects (Fay and Chase, 2009; Bernhardt and Edens-Meier, 2010). An orchid often relies on a single insect species for its pollination. Thus, maintaining a long floral life span is of great significance for the reproductive success of orchids. In fact, the FL of most members within the Orchidaceae is longer than those of other angiosperm species (Kerner von Marilaun, 1895), such as *Paphiopedilum dianthum*, which has an individual FL of 62 days, so that chances of pollination are high (Zhang et al., 2017). The long life spans of orchids are attributed to their floral mass per area (FMA) and the water maintenance characteristics of their flowers and leaves (Fu et al., 2012; Zhang et al., 2017). Investigating floral anatomy is crucial to understanding why Orchidaceae species usually have such long FL, because the anatomical traits of organs, such as leaves, may affect plant resource allocation, physiological functions, and adaptations to environmental changes (Holbrook and Putz, 1996; Vendramini et al., 2002; Pandey et al., 2009; Guan et al., 2011; Yang et al., 2018). However, the physiological and anatomical mechanism for long floral life span in orchids remains unclear.

The subfamily Cypripedioideae in Orchidaceae consists of five genera: *Paphiopedilum*, *Cypripedium*, *Mexipedium*, *Phragmipedium*, and *Selenipedium* (Cox et al., 1997; Cameron et al., 1999). *Paphiopedilum* and *Cypripedium* are closely related in phylogeny (Cox et al., 1997), with their flowers having a pouch-like lip, a shield-like staminode, a synsepal composed of fused lateral sepals, and two fertile stamens (Supplementary Figure 1), and some species of the two genera are pollinated by bees (Lindley, 1840; Cox et al., 1997; Liu et al., 2009; Guo et al., 2012; Chen et al., 2013). However, the life spans of the leaves and flowers of *Paphiopedilum* are longer than those of *Cypripedium* (Chang et al., 2011; Guan et al., 2011; Yang et al., 2018). The life span of each flower in *Paphiopedilum* is 26–62 days, depending on the species (Zhang et al., 2017), whereas that of *Cypripedium* is only 6–13 days (in this study). There are also other differences between the two genera. *Paphiopedilum* species are evergreen plants with fleshy leaves and which usually grow in karst limestone areas below an altitude of 2,000 m with a scarcity of soil and low water availability; plants in such areas often suffer from great water deficits, especially in the dry season (Cribb, 1998; Guan et al., 2011; Guo et al., 2012; Zhang et al., 2012; Yang et al., 2018). In contrast, *Cypripedium* species are deciduous plants with thin leaves that mainly grow in the shade of forests at altitudes above 1,800 m in southwest China (Cribb, 1997; Chen et al., 2005; Guan et al., 2011; Guo et al., 2012). The soil layer in habitats where *Cypripedium* species grow can store abundant water during the growing and flowering seasons. The differences in FL, morphology, habitat, and physiology between *Paphiopedilum* and *Cypripedium*, despite the close relatedness of the two genera, make them ideal candidates for exploring the association between FL and floral structure in orchids.

In this present study, we investigated the floral structural traits related to water balance and FL of 13 *Paphiopedilum* species and 5 *Cypripedium* species to explore the mechanism that affects the difference in FL between the two genera and test the evolutionary association between FL and floral structural traits related to water balance. We hypothesized that different floral life spans will represent different water-use strategies in the two closely related genera with different floral morphologies. Specifically, the *Paphiopedilum* species with longer floral life span may have stronger capacity to retain water in flowers to adapt to low-moisture habitats.

## Materials and Methods

### Plant Materials

We examined the association between FL and floral anatomy using 13 *Paphiopedilum* species and 5 *Cypripedium* species. The ecological characteristics, habitats, growth forms, and phenological periods of the 18 species are listed in Supplementary Table 1. The *Paphiopedilum* species were grown in a greenhouse at the Kunming Botanical Garden (102°410′E, 25°01′N; elevation 1,990 m). The growth conditions included an air temperature of 20–25°C during the day and 10–15°C at night and 60–70% relative air humidity. The *Cypripedium* species were grown at the Shangrila Alpine Botanical Garden (99°50′E, 27°48′N; elevation 3,260 m). The growth conditions included an air temperature of 15–24°C during the day and approximately 10°C at night and a relative air humidity ranging from 60 to 80% during the growing season.

### Floral Longevity

To investigate the FL of a single flower from each species of *Paphiopedilum* and *Cypripedium*, we randomly marked 3–20 newly emerged floral buds per species. Their individual flower opening and wilting dates were recorded throughout the flowering period. Each floral bud was sampled from a separate plant. The individual flower was identified as “opening” when the dorsal sepal rose and the visiting insects could enter the lip. The individual flower was regarded as “wilting” when the lip began to discolor, wilt, or was eaten by herbivores, resulting in the individual flower losing its ability to be pollinated (Sugiura et al., 2001; Zhang et al., 2017).

### Measurements of Flower Cross-Sectional Anatomy

Six individual flowers from six different plants per species were fixed in a formalin acetic acid–alcohol solution (37% formaldehyde, glacial acetic acid, 95% ethanol, and deionized water in a 10:5:50:35 mixture) for microscopic analysis. The samples (floral stem, petal, lip, dorsal sepal, and synsepal) were cleaned with water before anatomical analysis. Thin transverse cross-sections (20–30 μm) of the samples were made with a Microtome Cryostat (CM3050S; Leica, Germany); the sections were stained with 1% fuchsine for 4–5 s, mounted on glass microscope slides, examined, and photographed with a light microscope (U-CMAD3; Olympus Inc., Tokyo, Japan). Epidermal thickness; exodermal thickness; endodermal thickness of the floral stem; upper epidermal thickness; lower epidermal thickness; mesophyll thickness of the petals, lips, dorsal sepals, and synsepals; and the whole petal, lip, dorsal sepal, and synsepal thickness were determined with the ImageJ program.

### Measurements of Vein and Stomatal Densities

Vein density (mm mm^–2^) and stomatal density (mm^2^) were quantified from paradermal sections. The entire petals, lips, dorsal sepals, and synsepals were sampled and scanned at 2,400 dpi using a scanner. Vein density was measured as the total length of vascular tissue per mm^2^ of surface area, and stomatal density as the total number of stomata per mm^2^ of surface area, using the ImageJ software (National Institutes of Health, Bethesda, MD, United States).

### Statistical Analysis

Relationships among variables were analyzed using both pairwise Pearson and phylogenetically independent contrasts (PICs). The evolutionary associations were examined with PIC analysis by employing the “ape” package, utilizing molecular phylogenetic relationships (Cox et al., 1997; Li et al., 2011). A principal component analysis (PCA) was performed with the “prcomp” function of the “vegan” package to characterize the relationships among species or floral traits. Differences in floral traits between species of *Paphiopedilum* and *Cypripedium* were determined by the Mann–Whitney U test. All statistical analyses were performed with R software v. 2.15.0 (R Core Team, 2012).

## Results

All of the floral traits tested varied significantly across species, and the magnitude of variation differed for each trait (Table 1). The coefficients of variation (CVs) were >50% for endodermal thickness of the floral stem, mesophyll thickness of the petals, and stomatal density of the dorsal sepal and synsepal and <25% for the upper epidermis thickness of the dorsal sepal. Across all traits, stomatal density of the synsepal had the highest variation (93.18%), whereas vein density of the dorsal sepal had the lowest (21.62%).

**TABLE 1:** Quantification of floral structural traits for tested species.

Traits	Abbreviation	Unit	Functional significance	Mean ± SE	Min	Max	CV (%)
**Floral stem**							
Epidermal thickness	SEPT	μm	Water conservation	45.75 ± 1.18	19.32	77.67	26.71
Exodermal thickness	SEXT	μm	Water transport	325.76 ± 15.55	117.03	1,214.46	49.62
Endodermal thickness	SENT	μm	Water transport	136.09 ± 7.36	0	433.01	56.24
Petal							
Upper epidermal thickness	PUET	μm	Water conservation	67.38 ± 2.07	27.14	123.77	31.92
Lower epidermal thickness	PLET	μm	Water conservation	69.22 ± 2.24	26.46	128.82	33.57
Mesophyll thickness	PM	μm	Water storage	214.14 ± 10.79	55.56	483.50	52.37
Petal thickness	PT	μm	Water availability	348.77 ± 13.15	146.83	683.86	39.19
Vein density	PVD	mm mm^–2^	Water availability	1.11 ± 0.05	0.47	3.29	49.55
**Lip**							
Upper epidermal thickness	LUET	μm	Water conservation	65.87 ± 1.79	26.01	125.39	28.28
Lower epidermal thickness	LLET	μm	Water conservation	63.29 ± 1.70	25.04	121.38	27.98
Mesophyll thickness	LM	μm	Water storage	247.58 ± 10.33	34.80	540.03	43.38
Lip thickness	LT	μm	Water availability	375.53 ± 11.68	155.22	690.05	32.32
Vein density	LVD	mm mm^–2^	Water availability	0.93 ± 0.02	0.39	1.50	27.96
**Dorsal sepal**							
Upper epidermal thickness	DSUET	μm	Water conservation	64.38 ± 1.50	32.24	108.75	24.23
Lower epidermal thickness	DSLET	μm	Water conservation	74.81 ± 2.55	31.77	193.69	35.37
Mesophyll thickness	DSM	μm	Water storage	217.92 ± 8.81	16.37	478.23	42.00
Dorsal sepal thickness	DST	μm	Water availability	356.40 ± 10.43	149.24	632.32	30.40
Vein density	DSVD	mm mm^–2^	Water availability	1.11 ± 0.03	0.59	2.05	21.62
Stomatal density	DSSD	mm^–2^	Water loss	2.17 ± 0.13	0.55	6.77	61.75
**Synsepal**							
Upper epidermal thickness	SYUET	μm	Water conservation	61.06 ± 1.52	30.17	110.60	25.79
Lower epidermal thickness	SYLET	μm	Water conservation	64.75 ± 1.69	29.15	112.05	27.17
Mesophyll thickness	SYM	μm	Water storage	107.53 ± 4.01	17.11	238.25	38.73
Synsepal thickness	SYT	μm	Water availability	243.95 ± 5.90	130.67	452.58	25.15
Vein density	SYVD	mm mm^–2^	Water availability	1.22 ± 0.05	0.61	2.92	41.80
Stomatal density	SYSD	mm^–2^	Water loss	3.08 ± 0.28	0.56	11.29	93.18

**FIGURE 1:**
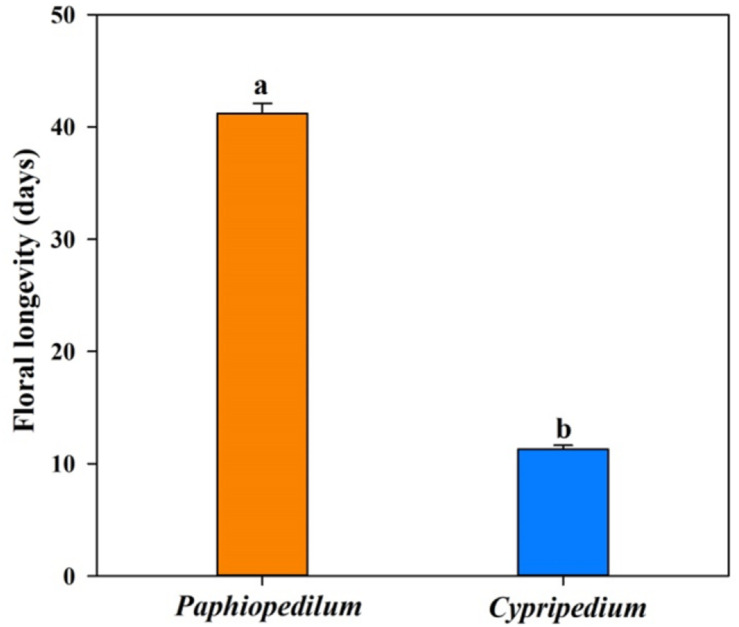
The floral longevity in *Paphiopedilum* and *Cypripedium* genera. SEs are presented with bars, and different letters signify statistically significant differences.

Twenty-four of the 25 floral anatomical traits and FL differed significantly between *Paphiopedilum* species and *Cypripedium* species (Figure 1 and Table 2). The former had longer FL and higher values for epidermis thickness (*P* < 0.001), exodermal (*P* < 0.001) and endodermal thickness (*P* < 0.001) of the floral stems, upper (*P* < 0.001) and lower epidermal thickness (*P* < 0.001), mesophyll thickness of the petals (*P* < 0.001) and dorsal sepal (*P* < 0.001), petal thickness (*P* < 0.001), lip thickness (*P* < 0.001), dorsal sepal thickness (*P* < 0.001), and synsepal thickness (*P* < 0.001), but lower values for vein (*P* < 0.001) and stomatal densities (*P* < 0.001) on the flowers. The values for mesophyll thickness of the lip (*P* = 0.128) and synsepal (*P* = 0.480) did not significantly differ between the two genera (Table 2).

**TABLE 2:** Contrasts in floral anatomical traits between *Paphiopedilum* and *Cypripedium* of floral functional traits for tested species.

Traits	*Paphiopedilum* (*n* = 13) Mean ± SE	*Cypripedium* (*n* = 5) Mean ± SE	*Z*	*P*
**Floral stem**				
Epidermal thickness	50.94 ± 1.09	32.26 ± 1.23	–7.31	<0.001
Exodermal thickness	371.05 ± 18.91	208.02 ± 9.05	–6.11	<0.001
Endodermal thickness	169.94 ± 6.88	48.07 ± 4.90	–7.85	<0.001
**Petal**				
Upper epidermal thickness	76.99 ± 1.90	42.40 ± 1.44	–7.83	<0.001
Lower epidermal thickness	79.28 ± 2.08	43.07 ± 1.95	–7.44	<0.001
Mesophyll thickness	252.24 ± 12.39	115.08 ± 4.30	–6.65	<0.001
Petal thickness	406.18 ± 13.16	199.49 ± 6.15	–7.83	<0.001
Vein density	0.88 ± 0.02	1.71 ± 0.13	–7.01	<0.001
**Lip**				
Upper epidermal thickness	72.82 ± 1.62	47.81 ± 3.00	–5.84	<0.001
Lower epidermal thickness	69.14 ± 1.58	48.07 ± 3.22	–5.29	<0.001
Mesophyll thickness	256.68 ± 11.74	223.94 ± 21.00	–2.52	0.128
Lip thickness	397.34 ± 12.68	318.84 ± 23.41	–2.79	0.005
Vein density	0.80 ± 0.02	1.26 ± 0.02	–7.94	<0.001
**Dorsal sepal**				
Upper epidermal thickness	70.66 ± 1.46	48.04 ± 1.56	–6.93	<0.001
Lower epidermal thickness	84.46 ± 2.77	49.73 ± 1.77	–6.94	<0.001
Mesophyll thickness	245.34 ± 10.27	146.64 ± 7.70	–5.70	<0.001
Dorsal sepal thickness	398.88 ± 10.64	245.95 ± 8.93	–6.79	<0.001
Vein density	0.96 ± 0.02	1.52 ± 0.06	–7.26	<0.001
Stomatal density	1.50 ± 0.07	3.90 ± 0.21	–7.62	<0.001
**Synsepal**				
Upper epidermal thickness	67.95 ± 1.40	43.14 ± 1.25	–7.70	<0.001
Lower epidermal thickness	72.50 ± 1.56	44.60 ± 1.39	–7.46	<0.001
Mesophyll thickness	107.71 ± 5.39	107.06 ± 3.62	–0.71	0.480
Synsepal thickness	262.96 ± 6.86	194.52 ± 4.67	–5.82	<0.001
Vein density	1.00 ± 0.02	1.79 ± 0.11	–6.57	<0.001
Stomatal density	1.59 ± 0.09	6.94 ± 0.50	–7.54	<0.001

**FIGURE 2:**
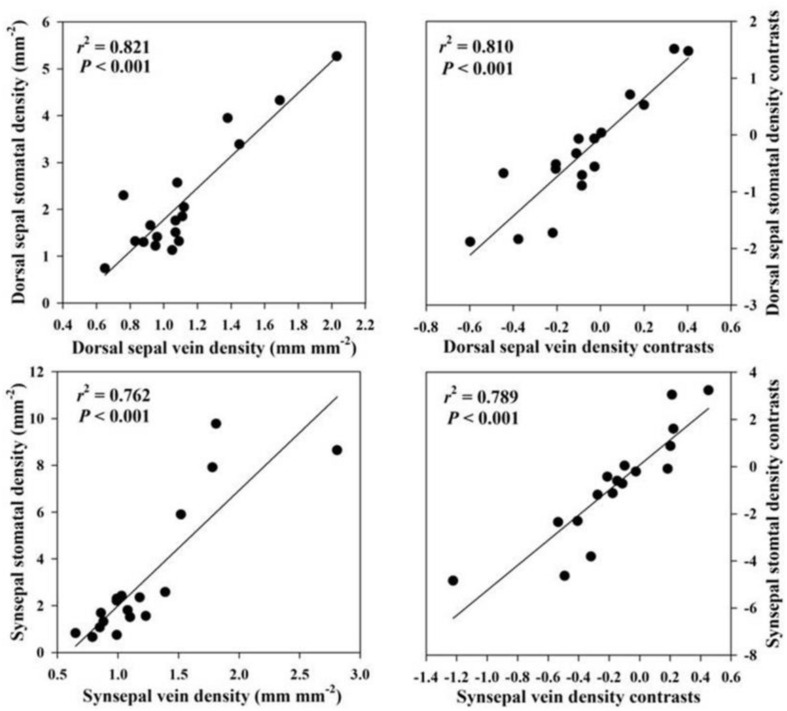
Correlations of vein density with stomatal density in the dorsal sepal and synsepal.

Across species, FL was positively correlated with epidermal thickness (*P* = 0.001) and endodermal thickness (*P* = 0.001) of the floral stems, upper (*P* = 0.007) and lower epidermal thickness (*P* = 0.006) of the petals, petal thickness (*P* = 0.036), vein density (*P* = 0.016) of the petals, upper (*P* = 0.042) and lower epidermal thickness (*P* = 0.038) and veins (*P* = 0.002) of the lips, upper (*P* = 0.004) and lower epidermal thickness (*P* = 0.002) of the dorsal sepal, vein (*P* = 0.001) and stomatal densities (*P* = 0.001) of the dorsal sepal, upper (*P* < 0.001) and lower epidermal thickness (*P* < 0.001), synsepal thickness (*P* = 0.011), and vein (*P* = 0.001) and stomatal densities (*P* < 0.001) of the synsepal. Moreover, these relationships were supported by using PICs (Table 3). No relationship was found between FL and other anatomical floral traits, such as exodermal thickness of the floral stem (*P* = 0.116), mesophyll thickness of the petals (*P* = 0.121), mesophyll thickness of the lip (*P* = 0.411), lip thickness (*P* = 0.214), and mesophyll thickness of the dorsal sepal (*P* = 0.06) and synsepal (*P* = 0.787). Similarly, these relationships were not found whether phylogeny was considered (Table 3). The FL was not correlated with dorsal sepal thickness when phylogeny was not considered (*P* = 0.161), but a significant correlation was found after phylogenetic correction (*P* = 0.035) (Table 3). The vein densities of the dorsal sepal (*P* < 0.001) and synsepal (*P* < 0.001) were positively correlated with stomatal density when a Pearson regression was used, and that correlation was still significant after correction (Figure 2).

**TABLE 3:** Pearson correlations and phylogenetically independent contrast (PIC) correlations for relationships between water-related traits with floral longevity in slipper orchids.

	Correlation with floral longevity		PIC	
Variables	*r*^2^	*P*-value	*r*^2^	*P*-value
**Floral stem**				
Epidermal thickness	0.526	0.001	0.520	0.0011
Exodermal thickness	0.166	0.116	0.153	0.120
Endodermis thickness	0.819	<0.001	0.825	<0.001
**Petal**				
Upper epidermal thickness	0.378	0.007	0.372	0.009
Lower epidermal thickness	0.382	0.006	0.401	0.006
Mesophyll thickness	0.144	0.121	0.226	0.054
Petal thickness	0.247	0.036	0.322	0.017
Vein density	0.312	0.016	0.249	0.042
**Lip**				
Upper epidermal thickness	0.234	0.042	0.249	0.041
Lower epidermal thickness	0.242	0.038	0.217	0.040
Mesophyll thickness	0.042	0.411	0.004	0.799
Lip thickness	0.094	0.214	0.037	0.457
Vein density	0.457	0.002	0.515	0.001
**Dorsal sepal**				
Upper epidermal thickness	0.415	0.004	0.438	0.004
Lower epidermal thickness	0.454	0.002	0.565	<0.001
Mesophyll thickness	0.060	0.327	0.129	0.158
Dorsal sepal thickness	0.161	0.099	0.263	0.035
Vein density	0.484	0.001	0.438	0.004
Stomatal density	0.491	0.001	0.717	<0.001
**Synsepal**				
Upper epidermal thickness	0.551	<0.001	0.556	<0.001
Lower epidermal thickness	0.635	<0.001	0.659	<0.001
Mesophyll thickness	0.005	0.787	0.073	0.295
Synsepal thickness	0.342	0.011	0.370	0.010
Vein density	0.506	0.001	0.536	<0.001
Stomatal density	0.610	<0.001	0.612	<0.001

**FIGURE 3:**
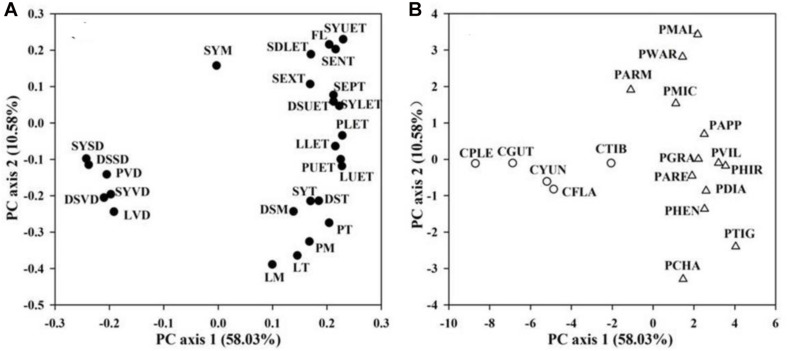
Principal component analysis (PCA) based on 26 floral traits **(A)** from 28 slipper orchids. PCA axes are presented in **(B)**. Values in parentheses along each axis indicate percentages of explained variation. Abbreviations for traits and species are shown in Table 1 and Supplementary Table 1, respectively.

In the PCA of floral traits, the first two PCAs explained 58.03 and 10.58% of the total variation, respectively (Figure 3). The first PCA axis was loaded by FL, floral functional traits of water conservation, transport, and storage (epidermis, exodermis, and endodermis thickness of the floral stems, upper and lower epidermis thickness of the petal, petal mesophyll thickness, petal thickness, upper and lower epidermis thickness of the lip, lip mesophyll thickness, lip thickness, upper and lower epidermis thickness of the dorsal sepals, dorsal sepal mesophyll thickness, dorsal sepal thickness, upper and lower epidermis thickness of the synsepal, synsepal mesophyll thickness, and synsepal thickness) on the positive side, and by floral functional traits of water supply and loss (vein and stomatal densities of the petal, lip, dorsal sepal, and synsepal) on the negative side (Figure 3). The second PCA axis was loaded by FL, the anatomical traits of the floral stem, dorsal sepal, and synsepal on the positive side, and by petal and lip anatomical traits, vein and stomatal densities of the petal, lip, dorsal sepal, and synsepal on the negative side (Figure 3). Species-loadings showed that the two genera, *Paphiopedilum* and *Cypripedium*, were well-separated along the first PCA axis. *Paphiopedilum* species were grouped on the positive side, whereas *Cypripedium* species clustered on the negative side (Figure 3), indicating that there was a significant difference in flower traits between the two closely related genera.

## Discussion

The present study supported our hypothesis that FL was tightly coupled with water conservation in flowers. The difference in FL between *Paphiopedilum* and *Cypripedium* was driven by floral anatomical traits related to reducing water loss, such as endodermal thickness and epidermis thickness of the floral stem and epidermis thickness, vein density, and stomatal density of the flower. Our findings provide new insights into the evolution of flowers in orchid plants.

Our results showed that FL was evolutionarily correlated with floral anatomical traits. The orchid species with longer FL had a thicker epidermis and endodermis of the floral stem and a thicker epidermis of the flowers, but a lower vein and stomatal density than species with shorter FL. This implied that the rate of water loss in flowers played an important role in regulating FL. FL can influence the total number of pollinators visiting a plant, thus affecting plant reproduction. At the same time, FL may reflect the balance between fitness consequences and maintenance costs (Kerner von Marilaun, 1895). Water loss represents a major drain for flowers with longer FL, and water stress can promote the accumulation of senescent-related substances in flowers, thus shortening the life span of flowers (Nobel, 1977; He, 1997; Wu and Zhao, 2001). Leaf life span is linked with leaf water-related traits, and trees with shorter leaf life spans have higher stem hydraulic efficiency and higher photosynthetic capacity and, thus, can achieve greater carbon gain in a shorter time (Fu et al., 2012). Previous research has also reported that stomatal limitations are associated with a long leaf life span (Brooks et al., 1997; Hikosaka et al., 1998). Stomata can rapidly respond to the change in the rate of water supply regulated by xylem conductance, thus reducing water loss and preventing xylem pressure from cavitation (Chaves et al., 2003). Zhang et al. (2017) found that FL is correlated with FMA and water maintenance traits in 11 *Paphiopedilum* species. This study extended our understanding of the role of floral anatomical traits in maintaining FL of slipper orchids and the critical application of anatomical traits as one of the key functional traits for maintaining FL.

The correlation of FL with anatomy likely reflects co-selection for water supply and loss. Across the species studied, our analyses showed that the strongest driver for the difference in FL was the endodermal thickness of the floral stem. The thicker endodermis was found in the floral stem of the *Paphiopedilum* species with longer life span, and there was a thinner or even no endodermis in the floral stem of *Cypripedium* species with shorter life span. The role of the endodermis is efficiently preventing water loss from the interior of the floral stem, allowing internal vascular bundles to transport water (Roppolo et al., 2011; Stasovski and Peterson, 2011; Qi et al., 2020). Moreover, the floral stem acts as a mechanical support (Enstone et al., 2003). The endodermis of plant with long FL tends to have a lower permeability and stronger support capacity than those species with short FL, possibly suggesting the adaptation of species with long FL to the requirements for conserving water, increasing water-use efficiency, and improving mechanical support in flowers during the flowering period in dry environments (Enstone et al., 2003; Roppolo et al., 2011).

We also found that FL was closely associated with epidermis thickness in flowers. This was shown by our Pearson’s and PICs analyses, which demonstrated that longer-lived flowers are more capable of maintaining flower turgor. The epidermis can function as a water conservation layer, and such flowers have a thicker epidermis, implying that they are more tolerant to drought stress (Guan et al., 2011; Zhang et al., 2012, 2015; Yang et al., 2018). However, there was no significant correlation between FL and mesophyll thickness (petal: *P* = 0.121; lip: *P* = 0.411; dorsal sepal: *P* = 0.099; synsepal: *P* = 0.787) (Table 3), indicating that FL might be determined by water conservation rather than water storage. Therefore, the increase in FL was mainly through reducing the water loss of flowers, rather than increasing the water storage capacity.

In leaves, water supply (vein density) is usually coordinated with water loss (stomata density) to keep the water balance (Brodribb and Jordan, 2011; Carins Murphy et al., 2012; Brodribb et al., 2013; Sack and Scoffoni, 2013; Wen et al., 2020). We found that the vein density in the dorsal sepal and synsepal was correlated with stomatal density, showing that this coordination was also mediated by the common dependence of vein and stomatal densities in flowers, and probably suggesting a functional similarity existed between the leaves and sepals in many plant species. The evolutionary correlation between vein density and stomatal density in sepals found in the present study was consistent with the previous study by Zhang F. P. et al. (2018).

Our results showed that there were clear distinctions in FL and floral anatomical traits between *Paphiopedilum* and *Cypripedium* species (Table 2). The former had significantly longer FL; thicker epidermis, exodermis, and endodermis of the floral stems; and had much thicker petals, lips, dorsal sepals, and synsepals, but lower vein and stomatal densities. These findings are in accordance with the results reported previously in leaves (Chang et al., 2011; Guan et al., 2011; Yang et al., 2018). The obvious divergences in FL and floral anatomical traits between the closely related genera, *Paphiopedilum* and *Cypripedium*, reflect the adaptation to their respective habitats. The natural growth conditions of *Cypripedium* species usually include nutrient-rich soil with high moisture content during the growing and flowering seasons. In contrast, the natural habitats of *Paphiopedilum* species in the karst limestone area are characterized by low water availability of leaking rocky substrates.

FL affects successful pollination of plants (Primack, 1985; Ashman and Schoen, 1994). The length of FL is an important mechanism for flowering plants to adapt to the richness of pollinators in specific habitats (Primack, 1985). Species with short-lived flowers are adapted to favorable weather conditions for pollinators to visit, whereas species with long-lived flowers are adapted to environments that are often not suitable for pollinators to visit (Kerner von Marilaun, 1895). In the habitat with rare pollinators and low activity of pollinator, a long FL can compensate for the shortage of pollinators to increase reproductive success (Ashman and Schoen, 1994; Bingham and Orthner, 1998; Fabbro and Körner, 2004; Vespirini and Pacini, 2005; Peng et al., 2012). Orchids often rely on specific pollinators (van der Pijl and Dodson, 1966). A “sit and-wait” strategy to increase FL is likely the only way for the flower to act its reproductive role in orchids (Kerner von Marilaun, 1895; Primack, 1985; Ashman and Schoen, 1994). Fruit set in slipper orchids (Cypripedioideae) with the trap-lip pollination system is highly variable even within the same species or same population in different years (Primack and Stacy, 1998; Zheng et al., 2010; Pemberton, 2013). A previous research showed that fruit set is correlated with FL in five *Cypripedium* species (Zheng et al., 2010). Fruit set of species in Cypripedioideae is often low, but the longer FL of the plants probably can compensate for rare pollinators (Pemberton, 2013). Thus, the long FL in *Paphiopedilum* might be explained as the result solely of a need for longer flowering time due to scarcity of pollinators in the habitats, whereas the favorable weather in the flowering period (from May to July) of species in *Cypripedium* is often suitable for pollinators to visit. Such favorable environments allow greater levels of pollinator visitation. Under such favorable conditions, flowers do not need to open for a long time to be visited by pollinators (Primack, 1985).

Flower represents a drain on the water of the plant as a result of transpiration and respiration (Nobel, 1977). A short FL may reduce the costs of maintaining flowers (Primack, 1985). In contrast, to maintain a long floral life span, flowers need to invest more physiological costs, such as water. Reducing water loss is important for maintaining the water balance of whole plants and flower turgor. Here, we found that *Paphiopedilum* species were characterized by higher values for floral traits related to water retention capacity. The special floral structures of *Paphiopedilum* might contribute to the maintenance of longer FL. These floral anatomical traits are important for flowers to be physiologically functional under low water availability conditions, as in leaves (Guan et al., 2011; Zhang et al., 2015; Yang et al., 2018). The differences in FL and floral anatomical traits between *Paphiopedilum* and *Cypripedium* indicated adaptations to their natural growing environments. These findings contribute to the conservation and cultivation of species in *Paphiopedilum* and *Cypripedium* and also provide a practical basis to predict flower water-stress tolerance for agriculturally important horticultural crops.

## Conclusion

In conclusion, our results supported the hypothesis that the cost of maintaining floral function measured by the floral anatomical traits was correlated with FL. The floral anatomical traits related to water conservation rather than water storage capacity contributed to the maintenance of FL in *Paphiopedilum* and *Cypripedium*. The divergence in FL and floral anatomical structures between these two closely related genera reflects the adaptations to their growing environments. Our findings provided strong evidence for functional association between FL and water conservation capacity in slipper orchids and highlighted the necessity of considering the water-use strategy of the reproductive organ when predicting global plant models.

## Data Availability Statement

The original contributions presented in the study are included in the article/Supplementary Material, further inquiries can be directed to the corresponding authors.

## Author Contributions

F-PZ, S-BZ, J-LH, WH, and HH designed the study. F-PZ, J-QF, and X-WF carried out the experiments. F-PZ analyzed the data. F-PZ and S-BZ wrote and revised the manuscript. All authors contributed to the article and approved the submitted version.

## Conflict of Interest

The authors declare that the research was conducted in the absence of any commercial or financial relationships that could be construed as a potential conflict of interest.

## Supplementary Material

Supplementary Figure 1 Typical floral structure of *Paphiopedilum appletonianum* **(A)** and *Cypripedium yunnanense* **(B)**.

Supplementary Table 1 The floral longevity and ecological traits for slipper orchids, *Paphiopedilum* and *Cypripedium*.
