# Supplementary material for: Floral Longevity of Paphiopedilum and Cypripedium Is Associated With Floral Morphology
Source: Front Plant Sci. 2021 May 31;12:637236. doi: 10.3389/fpls.2021.637236 (PMC8200665; doi:10.3389/fpls.2021.637236)
Supplement: Supplementary Figure 1 — Typical floral structure of Paphiopedilum appletonianum (A) and Cypripedium yunnanense (B). [file Data_Sheet_1.docx]

**SUPPLEMENTARY MATERIAL**


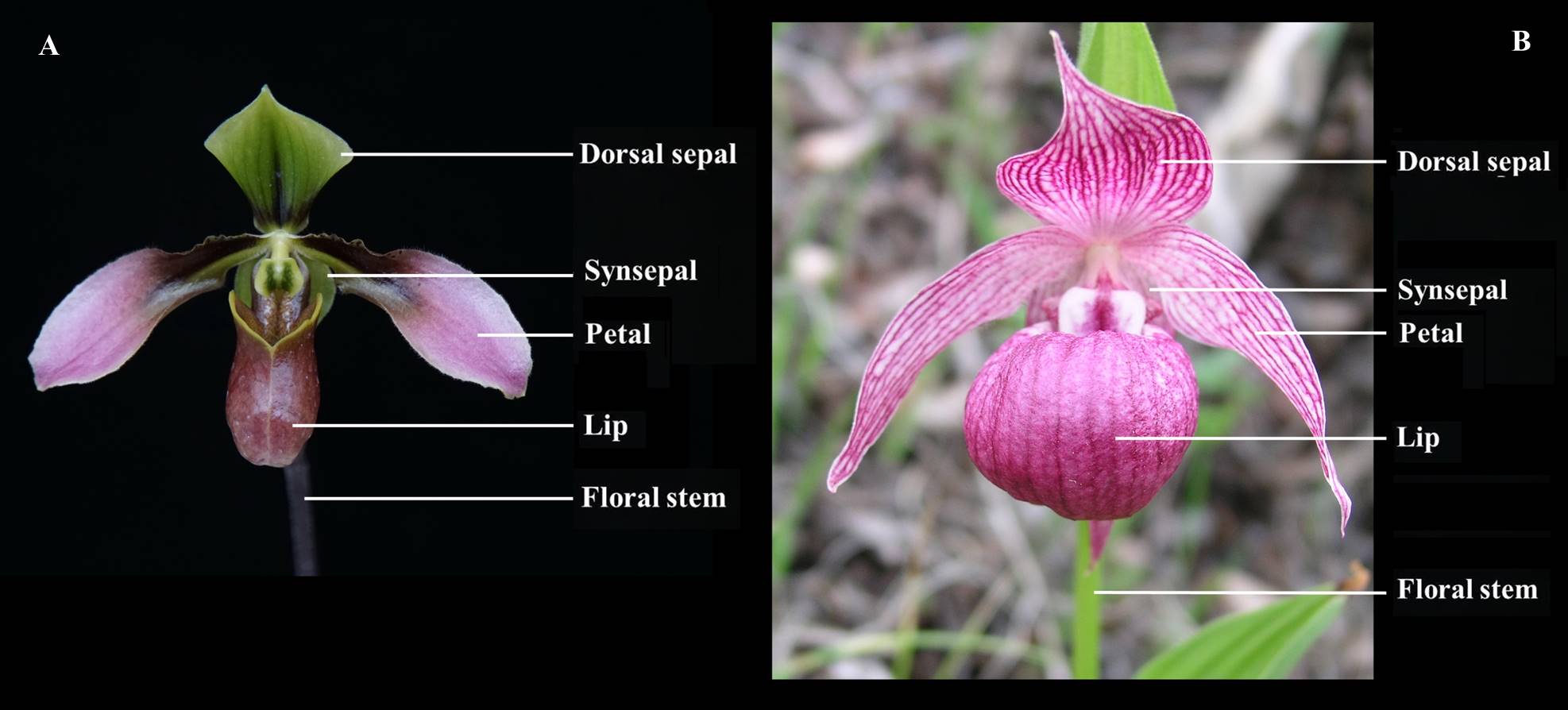


**Supplementary Figure 1∣**Typical floral structure of *Paphiopedilum appletonianum* (A) and *Cypripedium yunnanense* (B).

**Supplementary Table 1∣** The floral longevity and ecological traits for slipper orchids, *Paphiopedilum* and *Cypripedium*

| Species | Abbreviation | Floral longevity  （Mean ± SE）(days) | Leaf phenology | Habitat | Growth form | Altitude (m) |
| --- | --- | --- | --- | --- | --- | --- |
| *P*. *appletonianum* | PAPP | 53.47 ± 1.31 | Evergreen | In rocky and humus-rich places or crevices of rocks in forests | facultative | 300-1200 |
| *P*. *areeanum* | PARE | 38.00 ± 0.33 | Evergreen | On withered trees in forests | Terrestrial | 1000-1500 |
| *P*. *armeniacum* | PARM | 34.00 ± 0.63 | Evergreen | In rocky and well-drained places or in crevices of rocks | facultative | 1400-2250 |
| *P*. *charlesworthii* | PCHA | 26.00 ± 0.74 | Evergreen | In crevices on shady cliffs, in rocky and well-strained places of on rocks | Terrestrial | 1300-1600 |
| *P*. *dianthum* | PDIA | 62.13 ± 1.10 | Evergreen | On rocks in forests or thickets or shady cliffs | epiphytic | 1000-2250 |
| *P*. *gratrixianum* | PGRA | 41.00 ± 0.56 | Evergreen | Rocky places in forests | facultative | 1800-1900 |
| *P*. *henryanum* | PHEN | 32.07 ± 0.66 | Evergreen | In crevices of shady cliffs or rocky and well-drained places | facultative | 900-1300 |
| *P*. *hirsutissimum* | PHIR | 37.53 ± 0.51 | Evergreen | In crevices on shady cliffy, in rocky and well-strained places or on rocks | facultative | 300-1500 |
| *P*. *malipoense* | PMAL | 55.33 ± 1.09 | Evergreen | On glassy slopes or in humus-rich soil in forests and thickets | Terrestrial | 800-1600 |
| *P*. *micranthum* | PMIC | 28.45 ± 0.55 | Evergreen | In rocky and bushy places or crevices of rocks in forests | Terrestrial | 1000-1700 |
| *P*. *tigrinum* | PTIG | 37.27 ± 0.67 | Evergreen | On trees or mossy rocks or in rocky places in open forests | facultative | 1200-2200 |
| *P*. *villosum* | PVIL | 35.50 ± 0.81 | Evergreen | On trees or rocks in open forests | epiphytic | 1800-2200 |
| *P*. *wardii* | PWAR | 57.60 ± 0.49 | Evergreen | In grassy and bushy places on wooded slopes or forest margins | Terrestrial | 1200-2500 |
| *C*. *flavum* | CFLA | 13.06 ± 0.28 | Deciduous | Open forests and thickets, forest margins or stony and weedy slopes | Terrestrial | 1800-3500 |
| *C*. *guuttam* | CGUU | 6.80 ± 0.37 | Deciduous | Forests, thickets or grassland | Terrestrial | 500-4000 |
| *C*. *plectrochilum* | CPLE | 6.33 ± 0.88 | Deciduous | Stony and grassy slopes, forest margins | Terrestrial | 2000-3600 |
| *C*. *tibeticum* | CTIB | 10.89 ± 0.56 | Deciduous | Sparse forests, forest margins, scrubby and grassy slopes or stony places | Terrestrial | 2300-4200 |
| *C*. *yunnanense* | CYUN | 12.07 ± 0.40 | Deciduous | Open forests, thickets or grassy slopes | Terrestrial | 2700-3800 |
